# Detection of germline variants in human population chronically exposed to high level natural background radiation in Kerala coast

**DOI:** 10.1186/s41021-026-00352-4

**Published:** 2026-02-27

**Authors:** Vinay Jain, Divyalakshmi Saini, Radhakrishnan Sabarinathan, Birajalaxmi Das

**Affiliations:** 1 Low Level Radiation Research Section, Radiation Biology & Health Sciences Division, Bioscience Group, Bhabha Atomic Research Centre, Mumbai, 400085 India; 2 National Centre for Biological Sciences, Tata Institute of Fundamental Research, Bengaluru, 560065 India; 3 Homi Bhabha National Institute, Anushaktinagar, Mumbai, 400094 India

**Keywords:** Human population, Mutation spectrum, Low dose ionizing radiation (LDIR), Exome sequencing, Single nucleotide variations (SNVs), Indels, Chronic radiation, High-level natural radiation areas

## Abstract

**Background:**

Genetic effects due to long term exposure to low doses of ionizing radiation (LDIR) in humans are not well understood. Human population living in high level natural radiation areas (HLNRAs) of Kerala coast in India are continuously exposed to chronic LDIR emanating from monazite containing beach sand for many generations. The background radiation level in this area varies from < 1.0 to 45mGy/year. The people residing in HLNRAs sometimes receives background radiation dose which is approximately 10–40 times higher than the people living in adjacent normal level natural radiation areas (NLNRAs). This population provides a unique opportunity to identify, if present, a mutational signature due to chronic low-dose radiation exposure in humans. We have employed whole exome sequencing approach to determine germline mutational changes in the lymphocytes of healthy individuals from HLNRAs (mean background dose: 31.8 ± 5.4 mGy/year, mean age: 43.0 ± 5.9 years) and compared them with healthy individuals from NLNRAs (mean background dose: 0.9 ± 0.2 mGy/year, mean age: 43.0 ± 11.3 years).

**Results:**

Our results revealed that the overall number of single nucleotide variants (SNVs) and insertions/deletions (indels) were not significantly different in HLNRA (7744 SNVs, 880 indels) and NLNRA (7951 SNVs, 856 indels) groups. A similar number of protein affecting mutations (PAMs) were observed in HLNRA (1925) and NLNRA (2082) individuals. Interestingly, several unique SNVs were identified in both the groups. In HLNRA, unique SNVs were overrepresented in genes involved in important biological pathways such as DNA repair *(EXO1, PARP2, DDB1, POLQ, LIG1)*, epigenetic modification *(KDM5D, SETDB2, KMT2B, BRD8, SIRT1)*, cell cycle progression *(CDK14, CCND1*) etc. Furthermore, significant predominance of C > T transitions which were unique to HLNRA group was observed preferentially at CpG dinucleotide regions. Analysis with REVEL and AloFT tools did not show any increase in potentially pathogenic mutations including those involved in carcinogenesis in HLNRA individuals exposed to chronic radiation.

**Conclusion:**

This study did not show any significant changes in genetic variants due to long term exposure to LDIR in human population living in HLNRAs of Kerala coast. However, presence of unique SNVs and C > T transitions in CpG islands of HLNRA individuals indicate the possible role of epigenetic mechanisms i.e. DNA methylation in response to chronic LDIR in this population. This study significantly enhances the current understanding of radiation induced genetic changes and associated cancer risk in human population.

**Supplementary Information:**

The online version contains supplementary material available at 10.1186/s41021-026-00352-4.

## Introduction

Estimation of genetic risks in human population exposed to low dose ionizing radiation (LDIR) is a long-standing conundrum and highly relevant for understanding the impact of LDIR on human health. Human cells may accumulate mutations due to replication errors or unrepaired/mis-repaired DNA damage caused by various environmental exposures including ionizing radiation (IR). Spontaneous mutations in somatic cells may contribute towards induction of cancer, whereas mutations occurring in germ cells may contribute to heritable genetic diseases. Direct estimation of genetic risk due to LDIR depends upon mutations induced by IR as well as spontaneous *de novo* mutations in a population. Although, there is concrete evidence for radiation-induced mutations in different biological systems at higher doses, no such conclusive data on radiation-induced mutations leading to genetic diseases in humans is reported [1–4] In general, information on mutation spectrum in response to LDIR in human population is limited. At the same time, due to lack of direct human data on radiation-induced mutations, assumptions for risk estimation due to LDIR in humans is assessed by taking into consideration the induced mutation rate observed at high doses/dose rates in drosophila and murine model [1, 3]. Hence, it is highly essential to conduct studies on radiation exposed human population to establish the landscape of spontaneous or induced mutations, if any, due to LDIR. Therefore, human populations residing in high level natural radiation areas (HLNRAs) are ideal source to study mutational landscape associated with LDIR.

There are few HLNRAs in the world such as Kerala (India), Guarapari (Brazil), Yangjiang (China) and Ramsar (Iran), which provide information on the effect of LDIR on human population. But, the HLNRA of Kerala coast in south west India is unique for its vast and stable population with minimal migration, and a varying dose range (< 1.0 mGy to 45.0 mGy/year) due to patchy distribution of monazite content in the beach sand, which contains Uranium (0.8%), Thorium (8–10%) and their decay products [5–7]. HLNRA population are continuously exposed to elevated level of natural background radiation (mostly gamma radiation) and hence provide opportunity to study germinal mutation rates in human cells in response to LDIR.

The population residing in these areas provides unique opportunities to determine the spectrum of baseline mutations that is relevant to carcinogenesis directly on humans. It is important to estimate genetic risk based on germline or spontaneous mutations in the functionally active regions of the genome, which can be easily correlated with disease phenotypes (cancer and non-cancer diseases). Mutations that occur in protein coding regions are highly correlated to pathogenesis or diseased phenotype. However, epidemiological studies on genetic effect of IR in humans did not reveal any increase in congenital malformations or any type of adverse pregnancy outcomes in human [6–12]. Also, cancer incidences were not found to be increased in population living in HLNRAs of Kerala, India as well as Yangjiang, China [13–16]. No increase in hereditary anomalies and late onset non-cancer diseases are observed in HLNRA as compared to adjacent normal level natural radiation areas (NLNRA) [17, 18]. DNA damage analysis using different cytogenetic and molecular markers including phosphorylated histone H2AX at Ser139 (gamma-H2AX) and 53BP1 did not show background dose dependent increase in DNA double strand breaks (DSBs) in HLNRA individuals of Kerala coast [19–23]. However, few studies in HLNRAs of China and Ramsar, Iran have reported increased frequency of chromosomal aberrations in limited number of individuals studied [24, 25]. Recently, in contrary to previous reports, Aswathy et al. [26] have reported that the frequency of dicentric plus ring chromosomes in inhabitants of HLNRA individuals is higher than that of NLNRA individuals in Kerala coast. However, the contrasting results observed may be due to the fact that the study included only female inhabitants and it has been shown previously that cytogenetic endpoints such as dicentrics are known to exhibit inter-individual variability and can be influenced by lifestyle and sex-specific biological differences [27]. Most importantly, studies conducted in Kerala coast showed better and efficient repair of DNA breaks in HLNRA individuals [21, 28].

There are fewrecent reports where de-novo mutations have been studied in Chernobyl clean-up workers as well as Atomic Bomb (A-bomb) survivors. These studies did not show significant correlation between mutation frequency and LDIR [29–32]. However, no such study has been carried out in naturally exposed population living in HLNRAs.

Although no deleterious effects on LDIR are reported in humans yet, the Linear no-threshold (LNT) hypothesis, which states that cancer risk/health risk is directly proportional to radiation dose, remains the basis for regulatory safety and radiation protection science. LNT is based on the extrapolation of epidemiological data at high dose exposures from Atomic bomb survivors to low doses to establish cancer risks at LDIR [33, 34]. This hypothesis is highly debated since it does not take into consideration the DNA repair capacity of mammalian system which in-turn reduces mutational burden in the genome. It is also well established that DNA damage and repair process as well as mutation frequencies (germinal or somatic) are different at low and high doses of radiation exposure. Radiation induced mutation rate in humans is essential for risk estimation for doubling dose calculations but so far, it has been extrapolated from the germ line mutation rates from mice strains [2, 35]. Manifestation of mutations into pathogenesis and its consequences in terms of adverse health effects such as hereditary anomalies and cancer is a complex issue. Hence, it is important to establish mutation landscape due to LDIR in humans.

Radiation-induced genetic changes may occur non-randomly in a DNA molecule, mostly concentrate within “hotspot” regions of the genome [36]. Importantly, mutations in protein coding genes such as cancer predisposing and DNA repair genes etc. may initiate/promote the process of carcinogenesis. High-throughput sequencing of whole genome or exome provides insights into the spectrum and nature of genetic variations in the genome. Whole exome sequencing (WES) allows to decipher the genetic variations such as single nucleotide variants (SNVs), small DNA insertions or deletions (indels) and other structural variants (SVs) in the protein-coding region of the genome and has the advantages to identify novel variants/mutations in normal and diseased individuals. Hence, studying baseline mutation spectrum in healthy random donors are highly relevant for comparison of unique and common variants in a population [37].

The present study is focused on determining the mutation spectrum in individuals exposed to chronic high background radiation and matched controls from adjacent normal areas of Kerala coast using a high throughput exome sequencing approach. Attempt was also made to find out functional relevance of these mutations in response to chronic LDIR that is prevailing in HLNRAs of Kerala coast.

## Materials and methods

### Ethics statement and sample collection

Venous blood samples were collected by venipuncture from 12 random healthy male individuals from Kerala coast [NLNRA, (*N* = 6); HLNRA, (*N* = 6)] in EDTA vacutainers (BD™ Vacutainers, NJ, USA) in accordance with the methods and guidelines approved by Medical Ethics Committee, Bhabha Atomic Research Centre (BARC), Trombay, Mumbai. The written consent was obtained from all the participants along with information on confounding factors such as smoking, alcohol consumption, basic diseases, life style and medical radiation exposures using a standard questionnaire as per ICMR guidelines.

### Individual dosimetry

The external gamma radiation levels were measured in the house of each individual using halogen quenched Geiger Muller (GM) tube-based survey meter (Type ER-709, Nucleonix Systems, India). Both outdoor and indoor gamma measurements were done by placing dosimeter at a height of 1 m above the ground level. The annual absorbed dose (mGy/year) was calculated using a conversion factor of 0.0767 (= 0.8763 × 24 h × 365 days × 10^− 5^). The age and sex specific occupancy factor was used for the calculation of final radiation doses received by the individuals [19, 38]. The average dose received by NLNRA individuals (*N* = 6) was 0.9 ± 0.2 mGy/year and for HLNRA (*N* = 6) was 31.8 ± 5.4 mGy/year. The mean age among the individuals from both the group were similar (NLNRA; 43.0 ± 11.3 year and HLNRA; 43.0 ± 5.9 year).

### Separation of peripheral blood mononuclear cells from whole blood

Peripheral blood mononuclear cells (PBMCs) were isolated from the blood samples by density gradient centrifugation method using Histopaque-1077 solution (Sigma-Aldrich) as described elsewhere [19]. In brief, equal volume of blood was layered onto the Histopaque solution and centrifuged at 400 g for 30 min at room temperature. After centrifugation, buffy-coat layer containing PBMCs was separated and washed twice with freshly prepared chilled isotonic phosphate buffered saline (PBS) at 250 g for 10 min. PBMCs were processed with genomic DNA isolation as described below.

### Isolation of genomic DNA from PBMCs

Genomic DNA was isolated from PBMC using Purelink™ genomic DNA kit (K182001, Invitrogen, USA) according to the manufacturer’s procedure. The purity of isolated DNA was measured by taking ratio of absorbance at 260 nm and 280 nm using Nanodrop™ spectrophotometer. DNA concentration was measured using Qubit dsDNA HS Assay Kit (ThermoFisher Scientific, USA) in Qubit 3.0 Fluorometer (Invitrogen, USA). DNA integrity was checked on Bioanalyzer 2100 (Agilent Technologies, CA).

### Library preparation and sequencing run

The exome libraries were prepared from the DNA samples using TruSeq exome library preparation kit (Illumina, USA) as per manufacturer’s instructions. In brief, 100 ng of genomic DNA was fragmented in microTUBE™ pre-slit snap cap tubes (Covaris − 520045) using Covaris M220 instrument (Covaris, LLC, USA) according to Illumina protocol settings. Fragmented DNA was cleaned up and processed with end repair and size selection using sample purification beads. Adapters with unique indexes were ligated to sample specific libraries for pooling multiple samples before sequencing. The quality and size distribution of all the libraries were checked using High sensitivity DNA chip in Bioanalyzer 2100 (Agilent Technologies, USA). Libraries were quantitated using Qubit dsDNA HS Assay Kit (ThermoFisher Scientific, USA) in Qubit 3.0 Fluorometer (Invitrogen, USA) and pooled together before loading onto rapid flow cell. The libraries were sequenced in paired end mode (2 × 100 bp) on Hiseq 2500 platform (Illumina, USA). The sample homogeneity and quality of all FASTQ files were analyzed with FastQC tool [39].

### Mapping and variant calling

After quality control checks, the reads were mapped to the reference human genome (hg38) using bwa-mem through the Sarek pipeline v2.6.1 [40]. We obtained a mean coverage of ~ 60X in all samples (supplementary Table 1). The germline variants (SNVs and small indels) were called using Strelka (v2.9.10) and GATK Haplotypecaller (v4.1.7), following the GATK best practices. For Strelka, the variants that passed the default quality filters were selected. For GATK, we applied the following filters Quality by Depth (QD) < 2.0, Phred scaled quality score (QUAL) < 30.0, Strand Odds Ratio (SOR) > 3.0, Fisher Strand (FS) > 60.0, Mapping Quality (MQ) < 40.0, Mapping Quality Rank Sum Test (MQRankSum) < -12.5, Read Position Rank Sum Test (ReadPosRankSum) < -8.0 for SNVs; and QD < 2.0, QUAL < 30.0, FS > 200.0, ReadPosRankSum < -20.0 for indels. In addition, we required that the indels (predicted by Strelka or Haplotypecaller) should be supported by greater than 20 reads. Further, to remove polymorphic variants, we filtered out those SNPs and indels that overlapped in gnomAD [41] and genomeAsia100K [42] cohorts with minor allele frequency (MAF) > 1%. Finally, the variants that passed the above filters, and supported by both callers (that is, intersection of variants from both callers) were considered for further analysis.

### Analysis of protein affecting variants

To predict the functional impact of the variants, we used the variant effect predictor (VEP) tool. The variants annotated as missense, stop gained, frameshift, in-frame deletion, stop loss, or splice site, with the predicted functional impact to be high or moderate were defined as non-synonymous mutations (or protein affecting mutations). REVEL and AloFT tools were used to prioritize the potential pathogenic variants (missense and loss-of-function) [43, 44]. The variants predicted with a REVEL score of > 0.5 were considered pathogenic. In the case of ALoFT, we considered variants predicted to cause protein truncation with high confidence.

### Functional annotation analysis

Gene ontology and pathway analysis of protein affecting variants was carried out using open source pathway database Reactome (https://reactome.org/). To find out the cancer pre-disposition genes in our dataset, a comprehensive list of genes associated with cancer predisposition and DNA damage/repair was obtained from previous studies [45].

### Statistical analysis

Mann-Whitney test performed to assess significant difference between groups. Genes with an FDR adjusted p-value below 0.05 were considered significant. Principal component analysis (PCA) was performed for distribution of variants in the sample.

## Results

### Mutational landscape due to chronic low dose radiation

**Fig. 1:**
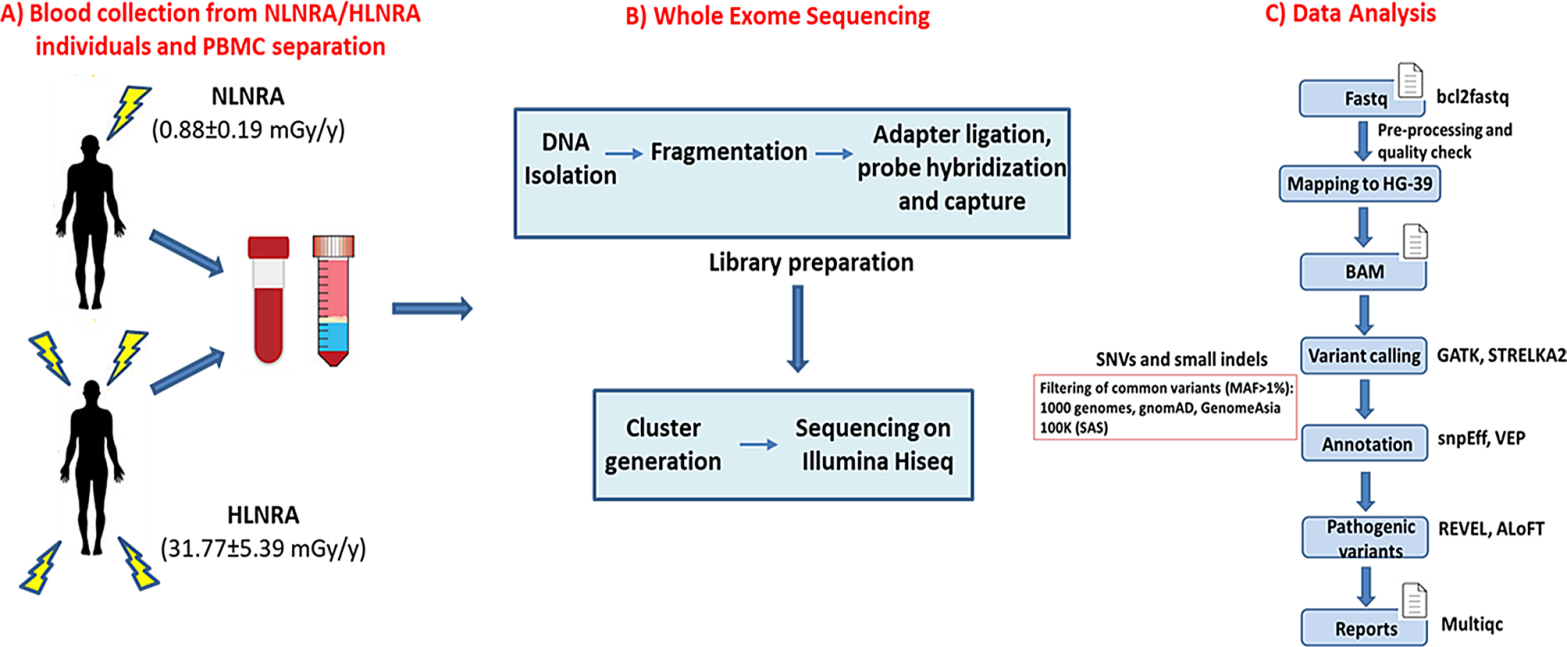
Schematic representation of the experiment design. (**A**) Blood sample collection from NLNRA and HLNRAs (**B**) Genomic DNA isolation and exome library preparation (**C**) Data analysis to identify SNVs and indels. NLNRAs: Normal level natural radiation areas, HLNRAs: High level natural radiation areas

Exome sequencing was carried out in order to determine the mutational changes due to long-term exposure to high levels of environmental radiation in HLNRA individuals. Matched controls from adjacent areas with normal levels of natural radiation (NLNRA) were included. The schematic representation of experiment design is shown in Fig. 1.

**Fig. 2:**
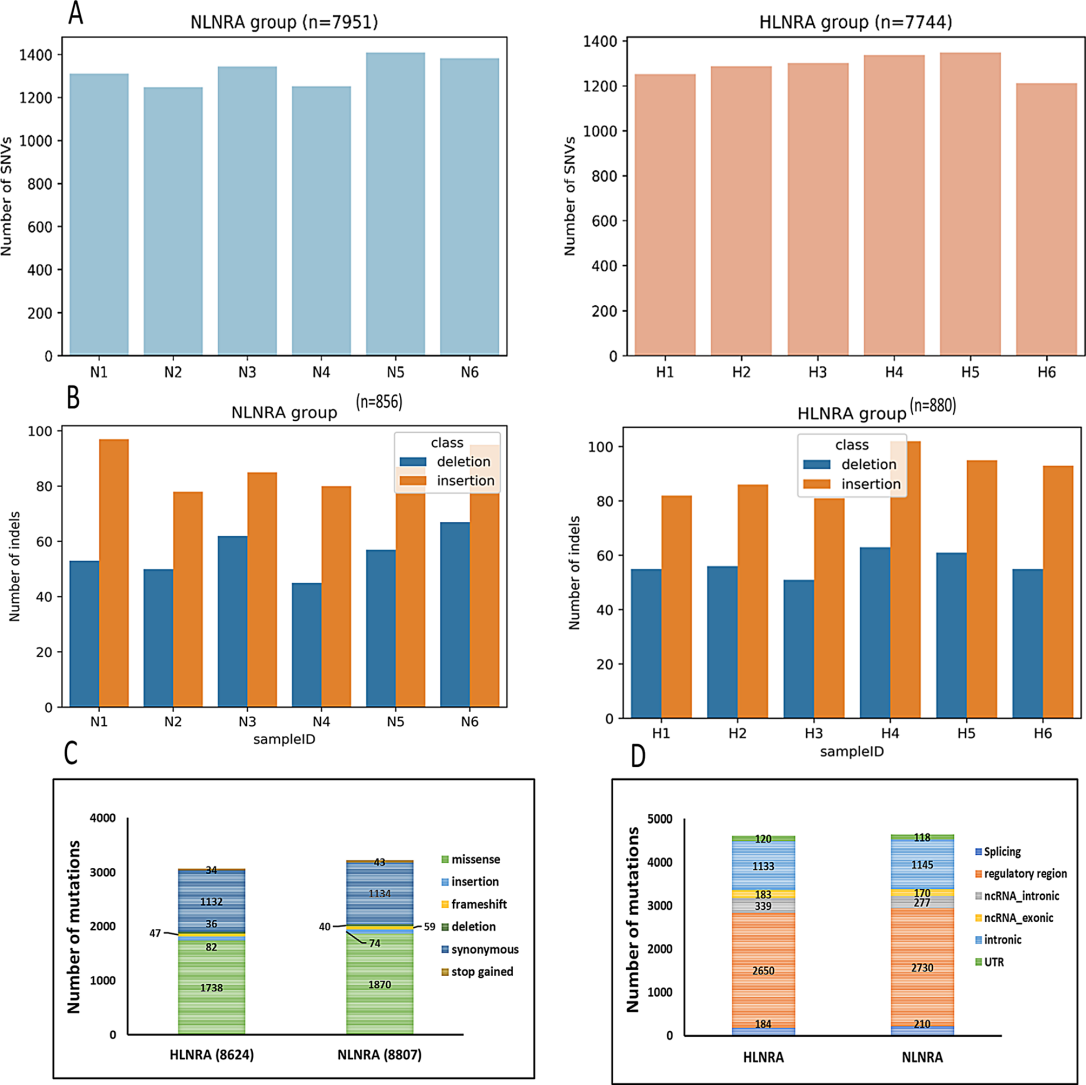
Mutation spectrum in NLNRA and HLNRA individuals. (**A**) Total number of SNVs in HLNRA and NLNRA groups. (**B**) Total number of indels in HLNRA and NLNRA groups. (**C**) Distribution of different types of mutations observed in HLNRA and NLNRA groups. (**D**) Representation of the genomic distribution of the mutations in both the group. NLNRAs: Normal level natural radiation areas, HLNRAs: High level natural radiation areas

Analysis was carried out to identify all the SNVs and indels present in HLNRA and NLNRA groups. We found 176,447 (Median SNV count: 29500) and 175,569 (Median SNV count: 29294) in NLNRA and HLNRA groups respectively (supplementary Fig. 1). We further filtered out the common polymorphisms (MAF > 1%) as reported in gnomAD [41] and genomeAsia100K [42] databases in order to identify mutational signature due to chronic background radiation. After filtration, only 17,431 variants were remaining, which consisted of 15,695 SNVs and 1736 indels. Of these, 8,807 mutations (7951 SNVs, 856 indels) were observed in NLNRA (control) group, whereas 8624 mutations (7744 SNVs, 880 indels) were found in HLNRA (exposed) group. Overall, the average number of SNVs observed among NLNRA (mean 1325 SNVs, 29.2 SNV per megabase) and HLNRA individuals (mean = 1290 SNVs, 28.4 SNVs per megabase) were found to be similar (Fig. 2A-B).

Further analysis was performed to find out the percentage of different types of mutations and their genomic distribution in NLNRA and HLNRA groups. Among the mutation types, non-synonymous (missense) mutations (HLNRA: 20.2%, *n* = 1738, NLNRA: 21.2%, *n* = 1870) were highest among both the groups. Around 13% synonymous (HLNRA: 13.1%, *n* = 1132, NLNRA: 12.9%, *n* = 1134) mutations were also observed (Fig. 2C). In terms of genomic distribution, maximum percentage of genomic variants were distributed in regulatory regions [~ 31%, HLNRA (*n* = 2650); NLNRA (*n* = 2730)]. Intronic regions constituted ~ 13% [(HLNRA; *n* = 1133; NLNRA (*n* = 1145)] in both the groups. Analysis also showed around 5% (*n* = 522) and 2.1% (*n* = 184) variants belonged to ncRNA and splicing region in HLNRA group as compared to 4% (*n* = 447) and 2.4% (*n* = 210) in NLNRA (Fig. 2D).

**Fig. 3:**
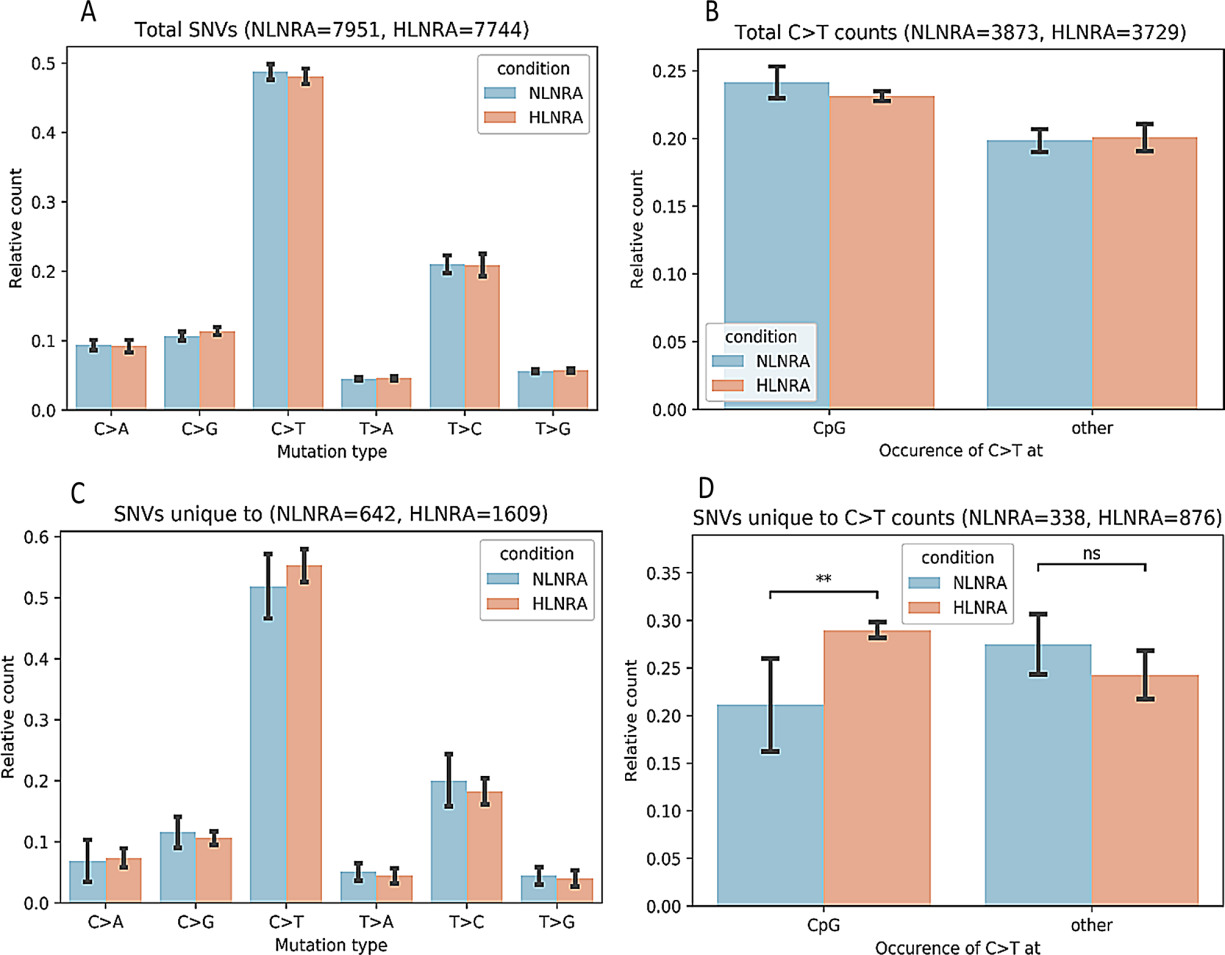
Distribution of single nucleotide variants (SNVs) in HLNRA and NLNRA groups. (**A**) Relative count of various types of SNVs in HLNRA & NLNRA groups. (**B**) Distribution of C > T mutation across different genomic regions. (**C**) Identification of unique SNVs (mutation types) among the groups. (**D**) Distribution of SNVs unique to C > T substitution in CpG and other genomic regions. NLNRAs: Normal level natural radiation areas, HLNRAs: High level natural radiation areas

Comparison of substitution types of mutations among SNVs between NLNRA and HLNRA group revealed that C > T mutation type (~ 50%) is the most predominant substitution type of mutations as compared to the other types (C > A, C > G, T > A, T > C and T > G). However, the frequency of C > T type of mutations (NLNRA = 3873 vs. HLNRA = 3729) was found to be similar between both the groups (Mann-Whitney U test, two-sided, *P* > 0.05). Moreover, the distribution of C > T mutations at the CpG sites and other regions are similar in both the groups (Fig. 3A and B). Further analysis was carried out on SNVs unique to each of the groups (NLNRA and HLNRA) present in at least more than one individual. SNVs exclusively found in NLNRA and HLNRA are 642 and 1609, respectively, but similar pattern of mutation types was observed, of which C > T mutations were found to be predominant type (Fig. 3C). But, in particular, the C > T mutation at CpG sites was significantly higher (two-sided Mann-Whitney U test, *P* = 0.005) in the HLNRA group as compared to the NLNRA group (Fig. 3D) in comparison to other regions.

**Fig. 4:**
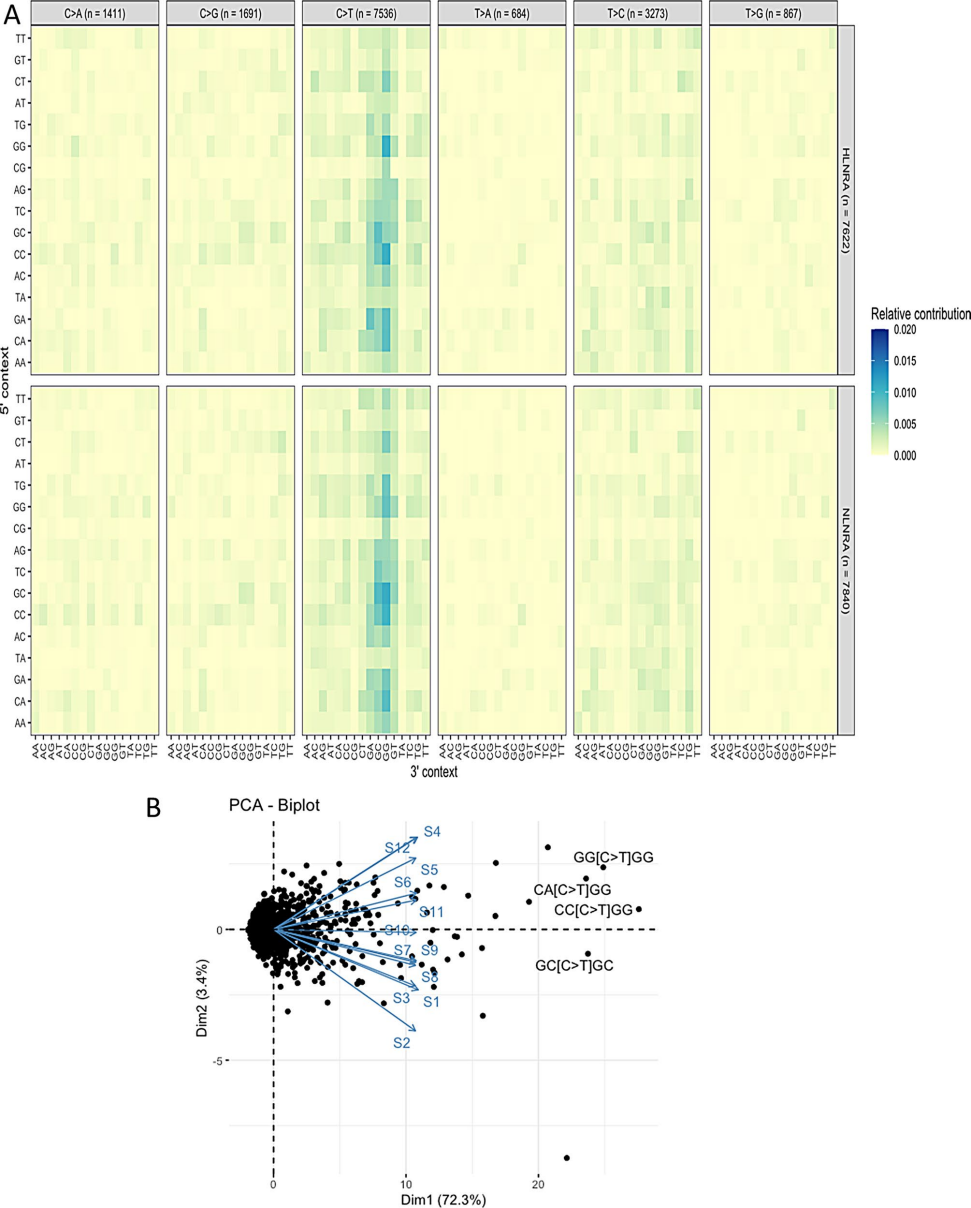
Analysis of variant frequency in the context of 5 mer (mutated position, along with two nucleotides upstream and downstream of the mutated position) to see the influence of sequence neighborhood (**A**) Heat map showing the effect of different dinucleotide combinations on various substitution mutations. (**B**) PCA analysis showing the influence of sequence combinations on C > T substitution. Individuals from NLNRA (control): ‘S1’, ‘S2’, ‘S3’, ‘S7’, ‘S8’, ‘S9’ and HLNRA: (‘S4’, ‘S5’, ‘S6’, ‘S10’, ‘S11’, ‘S12’). NLNRAs: Normal level natural radiation areas, HLNRAs: High level natural radiation areas

To check, whether the sequence neighborhoods other than dinucleotides is influencing the frequency of mutation types in both the groups, analysis of mutation frequency was computed in the context of 5-mer (mutated position, along with two nucleotides upstream and downstream of the mutated position). PCA analysis was performed which showed that the C > T mutations were also higher in HLNRA group in the context of CA[C > T]GG, CC[C > T]GG and GG[C > T]GG context (Fig. 4).

### Protein-affecting mutations in NLNRA and HLNRA groups

**Fig. 5:**
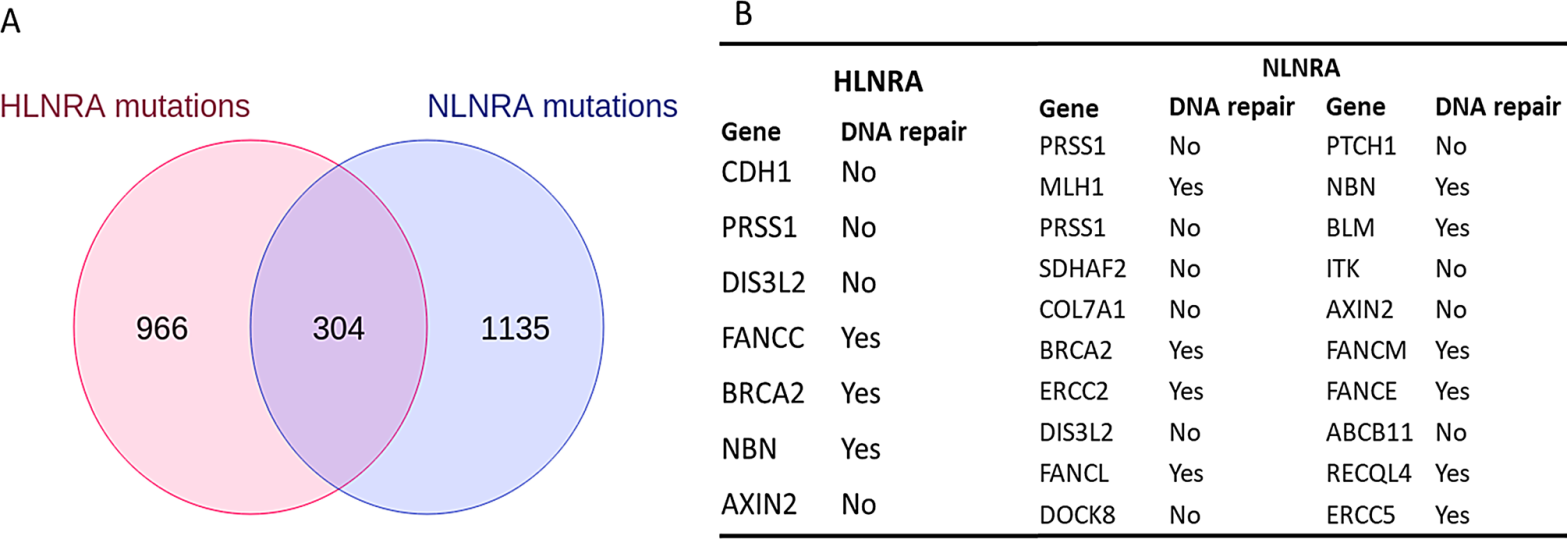
(**A**) Venn diagram showing number of genes (unique and common) with protein affecting mutations (PAMs) in HLNRA and NLNRA groups. (**B**) List of identified PAMs in cancer predisposing genes in HLNRA & NLNRA group. NLNRAs: Normal level natural radiation areas, HLNRAs: High level natural radiation areas

Ensemble variant effect predictor tool was used to predict the functional consequences of the mutations and compared between both the groups. Overall, the total number of proteins affecting mutations (PAMs) (such as missense, frameshift, inframe deletion, stop gained, splice donor and acceptor variants) were not different between the two groups (NLNRA:2082; HLNRA:1925). Venn diagram analysis revealed that a total of 966 and 1135 unique genes were carrying these mutations in HLNRA and NLNRA groups, respectively. Mutations in 304 genes were found to be common between both the groups (Fig. 5A and supplementary file 1).

Pathway analysis was carried out using reactome tool to find out biological functions / significance of these genes in both the groups. Interestingly, many genes involved in critical pathways such as DNA repair (HLNRA: *MRE11, LIG1, PARP2, MSH2, RAD9B, ATR* etc.; NLNRA: *FANCM, XRCC3, MLH1, ERCC1, ERCC5, FAN1* etc.), Immune response (HLNRA: *CNTFR, ITGB2, IL20RB* etc.; NLNRA: *IL20, IFI6, BCL6, CD44* etc.), chromatin organization/modifying enzymes (HLNRA: *SMARCC1, KDM5D, SETDB2, KMT2B, EP400, BRD8, SIRT1* etc.; NLNRA: *KANSL 1, BRPF3, SMYD2* etc.), RNA splicing (HLNRA: *PPIL1, HNRNPF, SNRPA* etc.,; NLNRA: *SNIP1, USP39, SNRNP35* etc.), cell-cell communication (HLNRA: *SDK1, CDH1, PTK2B* etc.,; NLNRA: *CLDN10, PRKC1, NECTIN1* etc.) were uniquely present in both the groups (Supplementary file 2).

We further analysed whether the cancer related genes are affected by PAMs. For this, we compared the PAMs in our dataset against a comprehensive list of cancer predisposing and DNA damage response genes (see Methods). We identified PAMs in 20 cancer disposing genes in NLNRA, whereas only 7 genes were found to be affected in HLNRA. Out of these, 5 genes (*PRSS1, DIS3L2, BRCA2, NBN, AXIN2*) were common in both the groups. However, *CDH1* and *FANCC* were unique to HLNRA group. Further, at least 10 cancer predisposition genes which are also involved in DNA repair were observed in NLNRA (*MLH1, ERCC2, FANCL, BLM, RECQL4* etc.) as compared to 3 genes (*FANCC, BRCA2* and *NBN*) in HLNRA (Fig. 5B).

Additionally, a total of 196 indels (NLNRA, 104; HLNRA, 92) were observed. Among them, 76 in-frame deletion mutations were observed (NLNRA, 40 and HLNRA, 36). Some of the common deletion mutations observed in both NLNRA and HLNRA were *DENND4B, GIGYF2, ZNF717, C18orf25, ALMS1, EPHB6, IFI27, NCOA3, GOLGA6L22, SMARCA2, ZNF714* and *WNK1*. Unique deletion mutations observed in NLNRA include *MCM2, IGSF3, CBX4, PPIG,* etc. Similarly, unique mutations observed in HLNRA were *CDK11A, ATXN1, DDB1, TAF15, RTF2, TRDN, MAGEC1, ZFHX3, FYCO1, MRPS28 and RSL1D1* (Supplementary file 1).

 Further analysis was carried out using REVEL and ALoFT tools to predict potential pathogenic missense and loss-of-function mutations (LoF) respectively in both the groups. In general, mutations with REVEL score of < 0.5 are considered as tolerable mutations (benign mutations) and mutations with cut-off > 0.5 are considered potentially pathogenic mutations. We identified around 145 and 187 mutations respectively in HLNRA and NLNRA group with a revel score of >0.5 (Supplementary File 3). Some of the important SNVs such as MLH1, POLG, PAX8, SMAD6, NDUFA9 etc. were unique in NLNRA. Similarly, unique SNVs observed in HLNRA include MRE11, CREB3, KMT2B, RADIL, BPNT etc. Top ten genes based on revel score > 9.0 is given in Table 1.

**Table 1:** List of genes carrying SNVs with highest Revel score in HLNRA and NLNRA groups

Chromosome	Position	Ref	Alt	Vep_gene	Vep_impact	Transcript_id	Protein_change	Group	Revel_score
chr9	125935573	A	G	PBX3	MODERATE	ENST00000373489	E270G	NLNRA	0.902
chr1	18634506	C	T	PAX7	MODERATE	ENST00000375375	R97W	NLNRA	0.907
chr19	10358032	C	A	TYK2	MODERATE	ENST00000525621	G761V	NLNRA	0.914
chr6	39868896	C	T	DAAM2	MODERATE	ENST00000398904	S279F	NLNRA	0.93
chr2	216447139	G	A	SMARCAL1	MODERATE	ENST00000357276	RG11H	NLNRA	0.931
chr12	4659103	G	A	NDUFA9	MODERATE	ENST00000266544	G160R	NLNRA	0.931
chr19	8103654	C	T	FBN3	MODERATE	ENST00000270509	C1616Y	NLNRA	0.932
chr12	102877530	T	A	PAH	MODERATE	ENST00000553106	I125F	NLNRA	0.95
chr3	37001037	A	G	MLH1	MODERATE	ENST00000231790	Y97C	NLNRA	0.951
chr7	92494382	C	T	PEX1	MODERATE	ENST00000248633	A981T	NLNRA	0.964
chr19	9982091	C	G	COL5A3	MODERATE	ENST00000264828	G812R	HLNRA	0.903
chr6	130899518	C	G	EPB41L2	MODERATE	ENST00000368128	M403I	HLNRA	0.905
chr3	136301034	C	T	PCCB	MODERATE	ENST00000469217	R317C	HLNRA	0.908
chr5	140364476	G	A	SLC4A9	MODERATE	ENST00000507527	R525H	HLNRA	0.911
chr19	48883821	C	T	TULP2	MODERATE	ENST00000221399	R403Q	HLNRA	0.917
chr6	49457989	C	T	MMUT	MODERATE	ENST00000274813	R152Q	HLNRA	0.921
chr16	67949842	A	G	SLC12A4	MODERATE	ENST00000422611	I571T	HLNRA	0.944
chr1	173909791	G	T	SERPINC1	MODERATE	ENST00000367698	P305H	HLNRA	0.956
chr7	127615429	C	T	PAX4	MODERATE	ENST00000378740	R39Q	HLNRA	0.962
chr7	92494382	C	T	PEX1	MODERATE	ENST00000248633	A981T	HLNRA	0.964

AloFT tool predicted 37 stop gain mutation with loss of function in NLNRA groups and 25 mutations in the HLNRA group with high VEP impact in our data set. The LoF mutations in *ZNF808, KALRN, RABEPK, CCDC7, TLCD2* etc., were exclusive to HLNRA group whereas *XRCC3, ATP2C2, TCF15, CHID1* etc. were exclusive to NLNRA group. However, *PRSS1, HLA-DRB5, PABPC3* were found to be common in both the groups (Supplementary file 3c).

### Highly represented unique mutations in protein coding genes in HLNRA and NLNRA groups

We further identified PAMs, that were exclusive to either of the groups (NLNRA or HLNRA) and present in at least two individuals within the group. A total of 903 (871 SNV and 32 indels) unique protein affecting mutations observed in HLNRA as compared to 371 (354 SNV and 17 indels) mutations in NLNRA group. Out of these 123 and 25 mutations were found in more than 50% of the individuals in HLNRA and NLNRA groups respectively. Some of the important protein coding genes with mutations were *ATR, HERC2*, *FANCA, BLK, MTERF3, POLR3H, RAD5 4 L, RADIL* (DNA repair), JPH3 (tumor suppressor gene), *RRBP1, RNF207, SNRPA* (Splicing/RNA binding), *CDK14, CCND1* (cell cycle progression), *EP400, BRD3, BPNT1, KMT2E, DNMT3B* (Epigenetic modification), *JPH1, GATA1, WNT8A, MAPK4* (signaling), *HSPA5, HSPA12B* (Protein folding), *CHST9* (cell-cell interaction), *HDDC2* (nucleotide metabolism). A complete list of mutations is given as supplementary file 4.

## Discussion

Evaluation of long-term consequences of chronic LDIR on human population is of utmost importance for risk estimation. Determining mutation frequency in a population is important for human health as these mutations may phenotypically manifest in the form of various diseases including cancer during later stages in life. Till today, consequences of long-term genetic effects of LDIR in humans are not well understood and chronic radiation induced mutational signatures are not identified. Due to lack of sufficient information on radiation effects in humans, a conservative model of risk estimation such as LNT hypothesis has been adopted by regulatory agencies, which considers even the smallest dose of radiation increases cancer/health risk in humans. The present study is focused on determining germline variants in blood samples of HLNRA individuals who were exposed to almost 30 times higher (mean dose: 31.8 ± 5.4 mGy/year) chronic background dose as compared to normal (control) group (0.9 ± 0.2 mGy/year. Notably, no adverse health effects in terms of congenital malformations and cancer is seen in HLNRA population exposed to such high levels of chronic natural background radiation [6–8, 11–18]. This study is aimed to provide novel insights on the changes in mutational landscape, if any, in response to high level natural background radiation in humans.

There are very limited reports available, where the frequency of germline mutations is studied in radiation exposed populations such as A-bomb survivors, Chernobyl clean-up workers, radiotherapy patients and naturally exposed population. Studies conducted in the children of A-bomb survivors using hypervariable markers such as mini and microsatellite did not show increased germinal mutation rates [30]. Comparable germinal mutation rate in mini- and microsatellites of newborns was observed between HLNRA and NLNRA of Kerala coast [29]. Also, no induction of germline mutations was reported in patients undergoing radiotherapy [46]. However, a study by Foster et al., showed increased mutation frequency in mitochondrial DNA in saliva samples collected from high background radiation areas [47].

In recent years, transgenerational studies done using WGS approach did not show significant increase in germline de-novo mutations in children born to Chernobyl clean-up workers as well as A- bomb survivors [30, 32]. Further, WES analysis has been extensively used to study mutation signatures in human cells exposed to non-ionizing radiation such as UV exposures in cancer cells [48, 49]. However, no WGS/WES based study to determine global germline or somatic mutation frequency in HLNRA population is available. In this study, we found similar frequencies of SNVs and indels in HLNRA (exposed) individuals compared with NLNRA (control) individuals. It is noteworthy to mention that these HLNRA individuals were exposed to > 30 times higher doses than control individuals. Any effect of confounding factors such as age and gender on mutation frequency was ruled out because all of the individuals studied were male and of similar age.

Accumulation of somatic mutations in CpG sites have been widely reported in different genomes. It is mostly attributed to frequent deamination of methylated cytosine into uracil, which leads to higher frequency of C to T mutations [50, 51]. Similar observations are found in our study in resting PBMCs of NLNRA and HLNRA individuals. Our data showed predominance of C > T mutations at CpG dinucleotides in both the population groups, although no significant difference was observed (*P* < 0.05) between the groups. However, an evident increase was observed in the frequency of uniquely present C > T mutations at CPG sites in HLNRA individuals. This could be an indication of differential methylation pattern at CpG islands in HLNRA individuals in response to LDIR. Our recent study on DNA methylation pattern of selected DDR and DNA repair genes in HLNRA have shown hyper-methylation at the promoter regions of some of these genes (unpublished). Similar findings were reported in promoter methylation analysis of MRE11A, PARP1, RAD23B gene in response to acute doses of IR [52].

The chronically exposed population of this area has been studied for spontaneous and induced DNA damage using cytogenetic assays, comet assay and gamma H2AX foci analysis and demonstrated efficient repair of DNA breaks [19–23, 38]. Transcriptome analysis in these individuals have revealed the activation of important genes involved in DNA damage response and repair, epigenetic mechanisms including DNA methylation, immune response, cell-cell communications and other survival pathways in HLNRA individuals [53]. It is important to note that cytogenetic assays detect gross chromosomal alterations, whereas whole-exome sequencing coupled with robust bioinformatics identifies base-level substitutions that may arise from epigenetic modulation, repair pathway variability, or radiation-adaptive processes. In this study, we found several protein affecting mutations (PAMs) in genes involved in these biological pathways, although the frequency of each type of mutations were similar in both the groups. Interestingly, our data showed unique mutations occurring in DNA repair, RNA splicing, immune response, methylation as well as chromatin modifying genes. For instance, mutations observed in DNA repair/epigenetic genes such as LIG1, MRE11, XRCC1, DDB1, KMT2B, EP400, BRD8, SIRT1 etc. in HLNRA group may be contributing towards better repair efficiency reported in HLNRA population. A separate study on SNP analysis also showed different genotypic frequency of DNA repair genes (NEIL1 and LIG1) in HLNRA and NLNRA population, which corroborates above observations [54].

Further, lower number of pathogenic PAMs were identified in HLNRA group. Only 7 cancer pre-disposing genes were shown to have PAMs as compared to 20 in NLNRA group. Out of these CDH1 and FANCC were unique to HLNRA group. Above findings are suggesting that LDIR has no adverse influence on genomic integrity and does not seem to increase mutational burden in the genome including in cancer pre-disposing genes in HLNRA. It may also be the plausible reason for not observing any increase in genetic effects including congenital malformations (birth defects and still birth) and excess relative risk (ERR) of any of the cancers in this population [16].

## Conclusion

In conclusion, the present study did not reveal any significant differences in the number of germline variants, including SNVs and indels, between individuals residing in NLNRA and HLNRA regions. These findings suggest the absence of detectable adverse genomic effects associated with long-term exposure to chronic low-dose ionizing radiation in the HLNRA population of the Kerala coast. Interestingly, the increased frequency of C > T transitions at CpG sites observed in the HLNRA group may reflect enhanced cytosine deamination and methylation-related processes. Such base-level modifications could represent an adaptive epigenetic response associated with long-term exposure to elevated natural background radiation. While these findings suggest a possible protective or compensatory mechanism operating in the HLNRA population, further investigations involving larger cohorts and genome-wide methylation analyses are required to substantiate this interpretation.

### Limitations and future perspective

This study is limited by the relatively small sample size in both NLNRA and HLNRA groups, partly due to the fact that individuals receiving background doses exceeding 20 mGy/year constitute only ~ 0.5% of the HLNRA population. As a result, the availability of participants from the highest exposure clusters is inherently restricted. Future studies incorporating larger cohorts and stratified dose categories will be critical for generating more definitive dose–response relationships and for identifying variants that may influence genomic stability under chronic low-dose radiation exposure. Furthermore, although high-depth whole-exome sequencing enabled detailed characterization of functional genomic regions, it does not capture the full spectrum of alterations that may occur in regulatory or non-coding regions. To achieve a more comprehensive understanding of radiation-associated genomic and epigenomic signatures, future investigations,—especially those focusing on individuals from the highest dose areas should employ whole-genome sequencing along with genome-wide methylation and epigenetic profiling. Such approaches will be instrumental in elucidating the molecular pathways that may underlie adaptive responses or protective mechanisms in populations chronically exposed to elevated natural background radiation.

## Supplementary Information

Below is the link to the electronic supplementary material.

Supplementary Material 1

Supplementary Material 2

Supplementary Material 3

Supplementary Material 4

Supplementary Material 5

Supplementary Material 6

## Data Availability

The data pertaining to the study is available on request to the corresponding author.
