# Supplementary figures and images for: Detection of germline variants in human population chronically exposed to high level natural background radiation in Kerala coast
Source: Genes Environ. 2026 Feb 27;48:5. doi: 10.1186/s41021-026-00352-4 (PMC12958757; doi:10.1186/s41021-026-00352-4)

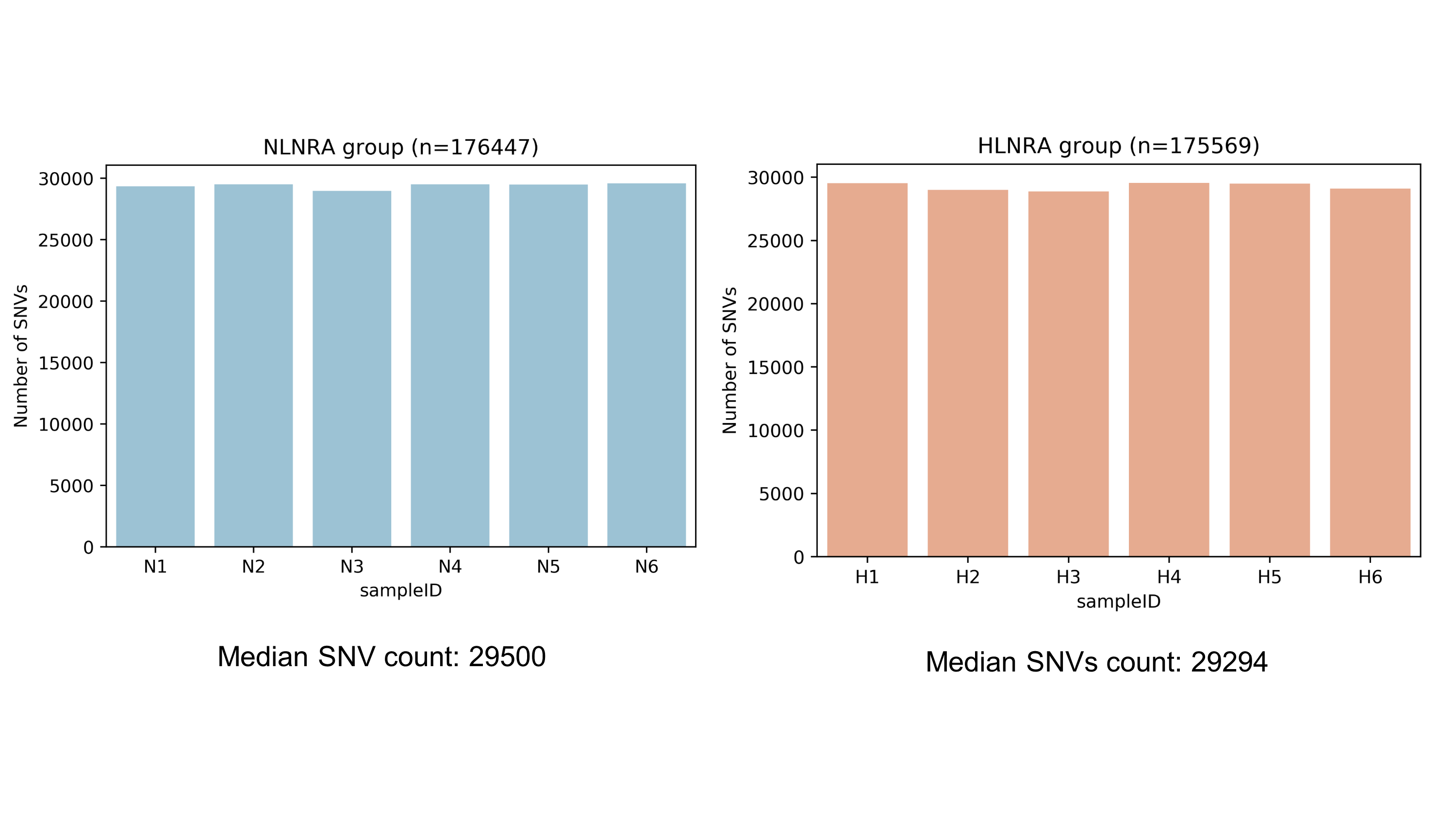

Supplement: Supplementary file 5 — Supplementary Material 5 [file 41021_2026_352_MOESM5_ESM.tif]
