# Supplementary material for: Detection of germline variants in human population chronically exposed to high level natural background radiation in Kerala coast
Source: Genes Environ. 2026 Feb 27;48:5. doi: 10.1186/s41021-026-00352-4 (PMC12958757; doi:10.1186/s41021-026-00352-4)
Supplement: Supplementary file 6 — Supplementary Material 6 [file 41021_2026_352_MOESM6_ESM.docx]

| **Supplementary table 1 : Exome sequencing reads, mean and average coverage of the samples studied from NLNRA (control) and HLNRA (exposed) groups of individuals.** | | | | | | | | |
| --- | --- | --- | --- | --- | --- | --- | --- | --- |
| **Sample name** | **Total reads** | **Mapped reads (global)** | **Percent mapped (global)** | **Mapped reads (on target)** | **Percent mapped**  **(on target)** | **Mean coverage (on target)** | **Group** | **Average Mean Coverage (X)** |
| **N1** | 47,386,031 | 47,274,651 | 99.76 | 35,818,317 | 75.59 | 61.9436 | NLNRA |  |
| **N6** | 50,546,492 | 50,441,368 | 99.79 | 40,275,256 | 79.68 | 69.5758 | NLNRA |  |
| **N4** | 44,096,313 | 44,019,719 | 99.83 | 35,516,408 | 80.54 | 61.9193 | NLNRA |  |
| **N5** | 38,286,357 | 38,221,281 | 99.83 | 31,013,629 | 81 | 52.7485 | NLNRA |  |
| **N3** | 44,384,208 | 44,265,651 | 99.73 | 34,328,891 | 77.34 | 59.4533 | NLNRA |  |
| **N2** | 43,905,889 | 43,792,806 | 99.74 | 33,835,131 | 77.06 | 58.7252 | NLNRA | 60.72761667 |
| **H3** | 44,504,254 | 44,369,258 | 99.7 | 33,594,027 | 75.48 | 57.5634 | HLNRA |  |
| **H1** | 38,440,692 | 38,363,560 | 99.8 | 29,977,364 | 77.98 | 51.8947 | HLNRA |  |
| **H6** | 48,381,166 | 48,276,436 | 99.78 | 37,408,943 | 77.32 | 63.8401 | HLNRA |  |
| **H2** | 46,432,317 | 46,309,413 | 99.74 | 35,700,099 | 76.89 | 61.2758 | HLNRA |  |
| **H4** | 53,106,727 | 53,023,149 | 99.84 | 41,918,379 | 78.93 | 71.0853 | HLNRA |  |
| **H5** | 44,229,073 | 44,139,132 | 99.8 | 35,292,504 | 79.79 | 60.8031 | HLNRA | 61.07706667 |
